# TNF-α and IL-1β-activated human mesenchymal stromal cells increase airway epithelial wound healing *in vitro* via activation of the epidermal growth factor receptor

**DOI:** 10.1186/s12931-015-0316-1

**Published:** 2016-01-11

**Authors:** Winifred Broekman, Gimano D. Amatngalim, Yvonne de Mooij-Eijk, Jaap Oostendorp, Helene Roelofs, Christian Taube, Jan Stolk, Pieter S. Hiemstra

**Affiliations:** Department of Pulmonology, Leiden University Medical Center, Albinusdreef 2, 2333 ZA Leiden, The Netherlands; Department of Clinical Pharmacy and Toxicology, Leiden University Medical Center, Leiden, The Netherlands; Department of Immunohaematology and Blood Transfusion, Leiden University Medical Center, Leiden, The Netherlands

**Keywords:** Lung, Chronic obstructive pulmonary disease, Inflammation, Airway epithelial cells, NCI-H292, Mesenchymal stromal cells, Wound healing, Regeneration, Repair, TNF-α/IL-1β

## Abstract

**Background:**

Mesenchymal stromal cells (MSCs) are investigated for their potential to reduce inflammation and to repair damaged tissue. Inflammation and tissue damage are hallmarks of chronic obstructive pulmonary disease (COPD) and MSC infusion is a promising new treatment for COPD. Inflammatory mediators attract MSCs to sites of inflammation and affect their immune-modulatory properties, but little is known about their effect on regenerative properties of MSCs. This study investigates the effect of the pro-inflammatory cytokines TNF-α and IL-1β on the regenerative potential of MSCs, using an *in vitro* wound healing model of airway epithelial cells.

**Methods:**

Standardized circular wounds were created by scraping cultures of the airway epithelial cell line NCI-H292 and primary bronchial epithelial cells cultured at the air-liquid interface (ALI-PBEC), and subsequently incubated with MSC conditioned medium (MSC-CM) that was generated in presence or absence of TNF-α/IL-1β. Remaining wound size was measured up to 72 h. Phosphorylation of ERK1/2 by MSC-CM was assessed using Western blot. Inhibitors for EGFR and c-Met signaling were used to investigate the contribution of these receptors to wound closure and to ERK1/2 phosphorylation. Transactivation of EGFR by MSC-CM was investigated using a TACE inhibitor, and RT-PCR was used to quantify mRNA expression of several growth factors in MSCs and NCI-H292.

**Results:**

Stimulation of MSCs with the pro-inflammatory cytokines TNF-α and IL-1β increased the mRNA expression of various growth factors by MCSs and enhanced the regenerative potential of MSCs in an *in vitro* model of airway epithelial injury using NCI-H292 airway epithelial cells. Conditioned medium from cytokine stimulated MSCs induced ERK1/2 phosphorylation in NCI-H292, predominantly via EGFR; it induced ADAM-mediated transactivation of EGFR, and it induced airway epithelial expression of several EGFR ligands. The contribution of activation of c-Met via HGF to increased repair could not be confirmed by inhibitor experiments.

**Conclusion:**

Our data imply that at sites of tissue damage, when inflammatory mediators are present, for example in lungs of COPD patients, MSCs become more potent inducers of repair, in addition to their well-known immune-modulatory properties.

## Background

In chronic obstructive pulmonary disease (COPD), the release of proteases and other mediators by a variety of inflammatory and resident cells is thought to cause tissue damage within the lung [1–3]. The endogenous regenerative capacity of the lung to restore damaged structures is limited, and the resulting imbalance between insufficient repair mechanisms and excess tissue damage will lead to irreversible tissue damage [4], ultimately causing organ failure.

Current COPD treatment targets symptoms, and there is a lack of treatments that halt disease progression and/or restore lung structure. The only current option for patients with chronic respiratory failure due to severe emphysema is lung transplantation, but the availability of donor lungs is limited and the success of lung transplantation varies. Therefore, new approaches to restore damaged lung tissue in COPD are needed.

A promising therapeutic approach that targets restoration of destructed lung tissue as well as reduction of inflammation is the administration of mesenchymal stromal cells (MSCs). MSCs are multipotent progenitor cells of non-hematopoietic origin defined by their capacity to differentiate into multiple lineages of the mesenchyme [5]. Besides their differentiation capacity, MSCs can favour repair of wounded tissue by modulating cellular responses in structural and immune cells, creating a regenerative and anti-inflammatory environment (reviewed in [6, 7]). The main mechanisms by which MSCs exert these effects are via cell-cell interactions and secretion of soluble factors.

Indeed, MSCs can reduce inflammation and repair alveolar structures as has been demonstrated in in vivo rodent models of cigarette smoke or elastase-induced emphysema [8–10]. It has been suggested that MSCs mediate this effect in part via the release of soluble factors, including hepatocyte growth factor (HGF) and epidermal growth factor receptor (EGFR) ligands, which can both increase proliferation of epithelial cells [11–15]. The receptors for these growth factors, c-Met and EGFR respectively, can activate extracellular signal-regulated kinase 1/2 (ERK1/2), one of the mitogen activated protein kinases (MAPK). Activation of this signaling pathway results in proliferation, differentiation and migration, processes that are fundamental for wound repair [16]. Whereas conditioned medium from MSCs has been shown to enhance airway epithelial wound healing *in vitro* [17], the contribution of HGF or EGFR ligands and underlying ERK1/2 signaling has not yet been investigated.

Another interesting and yet unanswered issue is whether pro-inflammatory cytokines can affect the potential of bone-marrow derived MSCs to repair damaged pulmonary epithelium at sites of inflammation. It is known that inflammatory mediators can attract MSCs (reviewed in [18, 19]) and alter their secretome [20–24], which is beneficial for the immune response [22] and for skin wound healing [25]. However, whether inflammatory mediators also increase the potential of bone marrow-derived MSCs to repair damaged pulmonary epithelium remains to be elucidated. Moreover, the cellular and molecular mechanisms that underlie such a repair potentiating effect within the airway epithelium are largely unknown.

Therefore, in the present study we investigated the effect of pro-inflammatory cytokines involved in the pathogenesis of COPD (i.e. Tumour Necrosis Factor-α (TNF-α) and Interleukin-1β (IL-1β)) [26–29] on the expression of growth factors by MSCs. We explored the effect of the conditioned medium from these stimulated MSCs on airway epithelial wound repair *in vitro*, and the contribution of the ERK1/2 signaling pathway, c-Met and EGFR to this effect. Our results show that stimulation of MSCs with TNF-α and IL-1β increases their regenerative potential as assessed in an *in vitro* model of airway epithelial repair. Furthermore, we demonstrate the crucial involvement of EGFR-activation in this process.

## Methods

### Cell culture

Cells from the NCI-H292 human lung mucoepidermoid carcinoma epithelial cell line (American Type Culture Collection, Manassas, VA, USA) were cultured in RPMI 1640 (Gibco, Grand Island, NY, USA), supplemented with 100 U/ml penicillin, 100 μg/ml streptomycin and 2 mM glutamine (all from Bio Whittaker, Walkersville, MD, USA) and 10 % [v/v] heat inactivated fetal calf serum (FCS) (Bodinco, Alkmaar, The Netherlands). Human primary bronchial epithelial cells (PBEC) isolated from tumour-free bronchial tissue [30] were cultured on semi-permeable transwell membranes with a 0.4 μm pore size (Corning Costar, Cambridge, MA, USA). Transwells were coated with 30 μg/ml PureCol (Advanced BioMatrix, San Diego, CA, USA), 10 μg/ml bovine serum albumin (BSA) (Sigma-Aldrich, St. Louis, MO, USA) and 10 μg/ml fibronectin diluted in PBS. Upon establishment of a confluent cell layer, PBEC were cultured at the air-liquid interface (ALI) during 2 weeks for differentiation. Culture medium consisted of a 1:1 mixture of bronchial epithelial growth medium (BEGM) (Lonza, Verviers, Belgium) and Dulbecco’s modified Eagle’s medium (DMEM) (Gibco), supplemented with 0.4 % (w/v) bovine pituitary extract (BPE), 1 μM hydrocortisone (HC), 0.5 ng/ml human epidermal growth factor (hEGF), 0.5 μg/ml epinephrine, 10 μ/ml transferrin, 5 μg/ml insulin, T3, 0.1 ng/ml retinoic acid (RA), 1 mM Hepes (all Lonza), 1 mg/ml BSA (Sigma-Aldrich), 100 U/ml penicillin and 100 μg/ml streptomycin (Lonza), and additional supplementation of 15 ng/ml RA (Sigma-Aldrich) for mucociliary differentiation.

Mesenchymal stromal cells (MSCs) were isolated from bone marrow from healthy donors and expanded in culture following a previously described protocol of the department of Immunohaematology and Blood Transfusion at Leiden University Medical Center [31]. MSC characterization was based on morphology and immunophenotyping using flow cytometry for the following markers: HLA-DR, CD73, CD90, CD31, CD34, CD45, CD80 (Becton Dickinson (BD) Bioscience, Franklin Lakes, NJ, USA) and CD105 (Ancell, Bayport, MN, USA), using FACSCalibur and CellQuest Pro Software (BD Bioscience). MSCs were cultured in DMEM GlutaMAX™ (Gibco), supplemented with 100 U/ml penicillin and 100 μg/ml streptomycin, and 10 % [v/v] heat-inactivated FCS (Thermo Fisher Scientific, UT, USA). All cells were cultured at 37 °C in a 5 % CO_2_ humidified incubator. Before experiments, NCI-H292 and MSCs were starved for growth factors overnight using serum-free (SF) culture medium; ALI-PBEC were starved for growth factors in B/D medium lacking BPE, HC, hEGF, RA and BSA. Prior to experiments, the apical side of ALI-PBEC cultures was washed with 100 μL of PBS to remove excess mucus.

### Preparation of conditioned medium

MSCs were grown until 80–90 % confluence and starved overnight in serum-free medium after washing with PBS. To generate MSC conditioned medium (MSC-CM), cells were cultured for 24 h in either serum-free medium (LG-DMEM) containing TNF-α and IL-1β (both 20 ng/ml; Peprotech, Rocky Hill, NJ, USA) (to generate MSC-CM^STIM^) or serum-free medium alone (to generate MSC-CM^CTRL^). MSC-CM from different donors and generated at different passages was pooled before experiments to reduce effects caused by donor and/or passage variation. All wound healing experiments were performed with MSC-CM at passage 4–6. A part of the western blot experiments were performed with MSC-CM at passage 2–4. Control DMEM medium with TNF-α and IL-1β (DMEM^STIM^) or without (DMEM^CTRL^) was obtained by incubating medium in culture flasks not containing cells in the same incubator for 24 h. MSC-CM and DMEM control medium were harvested and centrifuged for 7 min at 230xg to remove debris, and stored in 2 ml aliquots at -80 °C until further use.

### Wound repair model

A confluent monolayer of NCI-H292 cells or differentiated ALI-PBEC was mechanically injured by scraping the cell layer with a sterile Pasteur pipette with a soft tip (essentially as described in [32]). Two wounds with a diameter of 3 mm each were made in each well in a 12 wells plate for NCI-H292, or one wound per Transwelll insert for ALI-PBEC. Each experiment was performed in duplicate. After wounding, medium was replaced by the following stimuli: serum free standard culture medium (negative control); 20 ng/ml Transforming Growth Factor-α (TGF-α) (Sigma-Aldrich, St. Louis, MO, USA) in serum free standard culture medium (positive control [32]); or MSC-CM and the corresponding DMEM controls, which were all diluted 1:2 in serum free standard culture medium (based on dose response experiments), unless otherwise specified. For inhibitor experiments exploring the role of c-Met and the Epidermal Growth Factor receptor (EGFR) in wound healing, 0.05 μM PF04217903 (c-Met inhibitor; Sigma-Aldrich) and/or 0.2 μM AG1478 (EGFR tyrosine kinase inhibitor; Calbiochem, Darmstadt, Germany) were added to relevant stimuli for the full culturing period. Hepatocyte Growth Factor (HGF) (Peprotech) and TGF-α (both at 20 ng/ml in LG-DMEM) served as positive controls, whereas LG-DMEM was used as negative control (DMEM^CTRL^).

Digital images of the wounds were collected every 24 h up to 72 h maximum (NCI-H292) or at 6, 24 and 48 h (ALI-PBEC), on an inverted phase-contrast light microscope using Cell Sense Entry imaging software (both from Olympus, Tokyo, Japan). The surface of the wound area was measured using Image J software (National Institutes of Health, USA), and residual wound area in percentage was assessed by comparing the remaining wound size at different time points with the wound size at the start of the experiment ((1-wound size t = x/wound size t = 0)*100).

### Phosphorylation of ERK1/2

NCI-H292 cells used for assessment of ERK1/2 phosphorylation were cultured to 70 % confluence in a 12 wells plate. All stimuli were diluted 1:2 in RPMI. Cells were stimulated with either MSC-CM^STIM^ or DMEM^STIM^. HGF or TGF-α in a final concentration of 20 ng/ml diluted in LG-DMEM and DMEM^CTRL^ were used as positive resp. negative controls.

The role of c-Met and EGFR activation was investigated by pre-incubation of cells during 1 h with either 0.05 μM PF04217903, or 10 μM AG1478, or 2 μg/ml neutralizing antibodies against EGFR (Calbiochem) dissolved in RPMI in assigned wells before addition of stimuli. The role of transactivation was evaluated using the TNF-α converting enzyme (TACE)/ADAM17 inhibitor TAPI-1 (10 μM) (Santa Cruz Biotechnology, Dallas, TX, USA). Controls were treated with an equal volume of RPMI for 1 h.

Cells were incubated with stimuli for 15 min or various time periods for time-series experiments, and immediately cooled down on ice and washed with cold ERK washing buffer (5 mM Tris pH 7.4, 100 mM NaCl, 1 mM CaCl_2_, 1 mM MgCl_2_). Cell lysates were generated by incubation with lysis buffer (0.5 % [v/v] Triton X-100, 1 mM Na_3_VO_4_ and Mini complete protease cocktail (Roche, Basel, Switzerland) in ERK washing buffer) for 15 min. Samples from duplicate wells were pooled. After dilution in 2 times concentrated reducing sample buffer (4 % [v/v] SDS, 10 % [v/v] beta-Mercaptoethanol, 20 % [v/v] glycerol, 0.5 M Tris pH 6.8 and 0.003 % [w/v] Bromphenol blue) samples were boiled for 5 min and spun down at 20780xG for 5 min, before loading and running on a 10 % SDS-PAGE gel. Proteins were transferred onto a polyvinylidene difluoride membrane using the Mini-transblot system (both from Bio-Rad, Hercules, CA, USA).

Non-specific binding sites on the blots were blocked with PBS/0.05 % [v/v] Tween 20/0.5 % [w/v] casein (Sigma-Aldrich) for at least one hour followed by overnight incubation at 4 °C with antibodies directed against total and phospho-ERK1/2 (Cell Signaling, Beverly, MA, USA) in PBS/0.05 % Tween 20. After washing of the blot with PBS/0.05 % Tween 20, secondary HRP labeled goat antibodies (BD Bioscience) were added for 1 h at room temperature, followed by extensive washing. The membranes were developed on film (FujiFilm Corporation, Tokyo, Japan) using enhanced chemiluminescent (ECL) detection system (ThermoScientific, Rockford, IL, USA).

### mRNA expression

NCI-H292 cells were grown to near confluence and exposed to MSC-CM^STIM^ or DMEM^STIM^ or DMEM^CTRL^ (neg ctrl) 1:2 in RPMI. MSCs were grown to 80–90 % confluence and stimulated with TNF-α and IL-1β (20 ng/ml each) or serum free medium. Incubation times were based on initial experiments on limited samples revealing the largest increase of mRNA expression for several genes after 9 h (NCI-H292) and 6 h (MSCs) of stimulation. Sample triplicates from a 24 wells plate were pooled and RNA was extracted using Maxwell® 16 RNA Purification Kit (Promega, Madison, WI, USA) according to the manufacturers protocol, and quantified using the Nanodrop ND-1000 UV-visible spectrophotometer (Nanodrop Technologies, Wilmington, DE, USA). Complementary DNA was generated by adding Oligo(dT) primers (Qiagen, Düsseldorf, Germany) and 10 nM dNTP mix (Promega) to the RNA sample, and heating this to 65 °C for 5 min. Subsequently, 5x 1st strand RNA buffer, RNasin and M-MLV (all from Promega) were added, and the samples were incubated at 37 °C during 50 min followed by heat inactivation of M-MLV at 70 °C during 15 min. Primers were designed using PubMed Gene Database and Primerbank (http://pga.mgh.harvard.edu/primerbank) (Table 1) (Invitrogen, Thermo Fisher Scientific, Waltham, MA, USA). Quantitative real-time PCR was performed in triplicate in a 384 wells plate (Bio-Rad CFX384™), with samples mixed with respective primers and SYBR Green supermix (Bio-Rad) in a final volume of 8 μl. Results were checked for outliers: outliers were removed if the variance within triplicates was above 10 %.

**Table 1:** Primers for RT-PCR

GENE	Primer sequence FW	Primer sequence RV
*ACTB*	TTC CAG GAG CGA GAT CCC T	CAC CCA TGA CGA ACA TGG G
*ATP5B*	TCA CCC AGG CTG GTT CAG A	AGT GGC CAG GGT AGG CTG AT
*GAPDH*	TTC CAG GAG CGA GAT CCC T	CAC CCA TGA CGA ACA TGG G
*RPL13A*	AAG GTG GTG GTC GTA CGC TGT G	CGG GAA GGG TTG GTG TTC ATC C
*AREG*	GGT GGT GCT GTC GCT CTT G	AGG TGT CAT TGA GGT CCA ATC C
*CCDN1*	CAA TGA CCC CGC ACG ATT TC	CAT GGA GGG CGG ATT GGA A
*EGF*	TGC AGA GGG ATA CGC CCT AA	CAA GAG TAC AGC CAT GAT TCC AAA
*FGF2*	TGG CTA TGA AGG AAG ATG GAA GA	TCC AAT CGT TCA AAA AAG AAA CAC
*HB-EGF*	TGG ACC TTT TGA GAG TCA CTT TAT CC	CGT GCT CCT CCT TGT TTG GT
*HGF*	TCC AGA GGT ACG CTA CGA AGT CT	CCC ATT GCA GGT CAT GCA T
*IL6*	CAG AGC TGT GCA GAT GAG TAC A	GAT GAG TTG TCA TGT CCT GCA G
*PDGFA*	CAC CAC CGC AGC GTC AA	CCT CAC CTG GAC TTC TTT TAA TTT TG
*TGFA*	AGG TCC GAA AAC ACT GTG AGT	AGC AAG CGG TTC TTC CCT TC
*VEGF*	CGA GGG CCT GGA GTG TGT	TGG TGA GGT TTG ATC CGC ATA

Expression of *ACTB* and *GAPDH* was used to normalize mRNA expression in MSCs, whereas *RPL13A* and *ATP5B* were used for NCI-H292 (Table 1).

#### Statistics

The data are expressed as mean ± standard error of the mean unless depicted otherwise. GraphPad Prism 6.0 (GraphPad Inc., La Jolla, CA, USA) was used for statistical analysis. For comparison between groups, the Mann-Whitney test was used, for comparison of three or more groups the Kruskall Wallis test. Differences were considered significant when *p* < 0.05.

## Results

### Stimulation of MSCs with pro-inflammatory cytokines induces mRNA expression of several growth factors and leads to increased protein levels of HGF

To investigate the effect of pro-inflammatory cytokines on growth factor expression in MSCs, the mRNA expression of a variety of growth factors was analyzed in MSCs stimulated with TNF-α and IL-1β (20 ng/ml each).

**Fig. 1:**
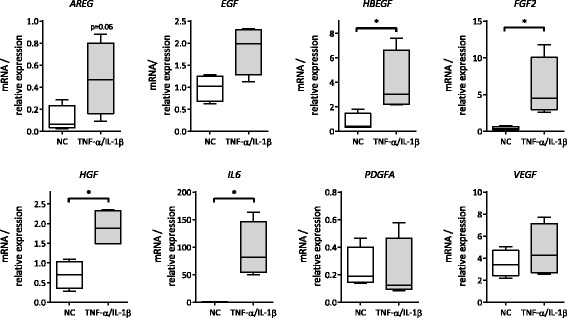
Stimulation of MSCs with TNF-α and IL-1β increases the expression of several growth factors. MSCs were stimulated with TNF-α and IL-1β 20 ng/ml each and harvested for RNA extraction after 6 h. mRNA expression of various growth factors (*AREG*, *EGF*, *HBEGF* (all EGFR ligands), *FGF2, HGF, IL6, PDGFA*, and *VEGF*) was determined by qPCR, which showed a significant increase of *FGF2*, *HBEGF*, *HGF* and of *IL6*, and an increase of *AREG* and *EGF*. Values were normalized to *ACTB* and *GAPDH* reference genes. Box and whiskers represent median, interquartile range and minimum and maximum for *n* = 4 obtained from three different donors; (*) *p* < 0.05

The mRNA expression of the growth factors Fibroblast Growth Factor 2 (*FGF2*), Hepatocyte Growth Factor (*HGF*), Heparin-binding EGF-like growth factor (*HBEGF*) and Interleukin-6 (*IL6*) was significantly increased (*p* < 0.05), and a non-significant increase was observed in amphiregulin (*AREG*) (*p* = 0.06). mRNA expression of the other growth factors evaluated did not change significantly (Fig. 1).

### MSC conditioned medium increases wound closure in NCI-H292 monolayers

Exposure of MSCs to the pro-inflammatory cytokines TNF-α and IL-1β resulted in increased mRNA expression of several growth factors. To assess whether this observation has functional relevance, a wound closure model was used to investigate the effect of MSC-CM^STIM^ on wound closure in NCI-H292 airway epithelial cell monolayers.

**Fig. 2:**
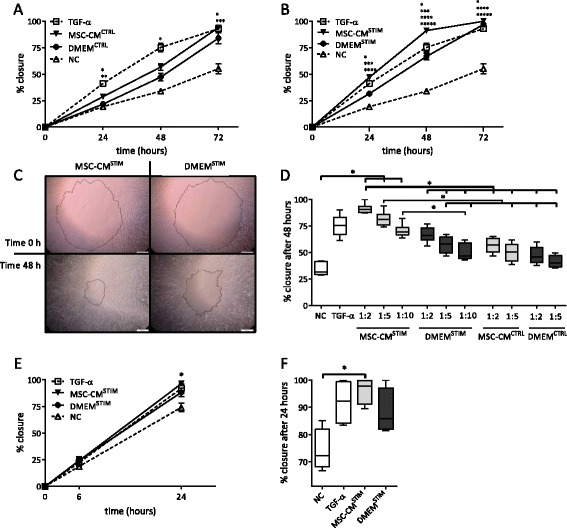
MSC-CM increases wound closure. **a**, **b**: NCI-H292 cells were injured by making a circular wound with a diameter of 3 mm, and subsequently incubated with MSC-CM^CTRL^, MSC-CM^STIM^, DMEM^CTRL^ and DMEM^STIM^, and a negative control (NC, RPMI only) and positive control (RPMI supplemented with TGF-α 20 ng/ml). The wound size was measured at 0, 24, 48 and 72 h after wounding. MSC-CM^STIM^ significantly increased wound closure. The effect of MSC-CM^STIM^ was dose-dependent. Error bars represent standard error of the mean (SEM). *n* = 4-6; (*) *p* < 0.05 TGF-α compared to NC and (**) compared to DMEM; (***) *p* < 0.05 MSC-CM compared to NC and (****) compared to DMEM; (*****) *p* < 0.05 DMEM^STIM^ compared to NC. **c**: Morphology of the closure of the wound: photos in the upper panel are taken at t = 0 h, the lower panel shows the same wounds photographed 48 h later. The two photos on the left side are obtained from MSC-CM^STIM^ stimulated cells, whereas photos at the right represent its control, DMEM^STIM^. **d**: Dose response. Box and whiskers represent median, interquartile range and minimum and maximum. *n* = 4-6; (*) *p* < 0.05. **e**: Wound closure in ALI-PBEC measured at 6 and 24 h (at 48 h all wounds were closed). At 24 h, MSC-CM^STIM^ significantly enhanced wound healing compared to the NC, but no significant differences were observed compared to its control, DMEM^STIM^. Error bars represent SEM, *n* = 4; (*) *p* < 0.05 for MSC-CM^STIM^ compared to NC. **f**. Wound closure in ALI-PBEC at 24 h. Box and whiskers represent median, interquartile range and minimum and maximum, for *n* = 4 as in Fig. 2f; (*) *p* < 0.05

No significant differences were observed when wounded NCI-H292 cells were incubated with conditioned medium from unstimulated MSCs (MSC-CM^CTRL^), compared to the control medium (DMEM^CTRL^) (Fig. 2a). In contrast, MSC-CM from TNF-α and IL-1β stimulated MSCs (MSC-CM^STIM^) significantly enhanced wound closure compared to DMEM^STIM^ (also containing TNF-α and IL-1β) after 24 and 48 h and was even more effective than the positive control TGF-α (Fig. 2b, c) (*p* = 0.002 at 48 h). The effect of MSC-CM^STIM^ was dose-dependent and still detectable at a 1:10 dilution (Fig. 2d).

Next the effect of MSC-CM on wound closure in well-differentiated cultures of ALI-PBEC was investigated. Wound closure in ALI-PBEC cultures was faster than in NCI-H292, and full wound closure was observed within 48 h. In ALI-PBEC, MSC-CM^STIM^ also significantly increased epithelial wound closure, however to a similar extent as its control (DMEM^STIM^) (Fig. 2e, f).

### MSC-CM^STIM^ activates ERK1/2 signaling in NCI-H292 monolayers via (trans)activation of the EGF-receptor

The observation that MSC-CM^STIM^ enhanced wound closure in NCI-H292 monolayers prompted further investigation into the underlying cellular response accountable for this effect. Epithelial wound healing is regulated by activation of mitogen activated protein kinases (MAPKs), and in particular via activation of the MAPK Extracellular signal-Regulated Kinase (ERK)1/2, which is known to be involved in cell proliferation, differentiation and migration [16, 33]. Therefore we further examined the role of this pathway in MSC-CM induced wound healing.

**Fig. 3:**
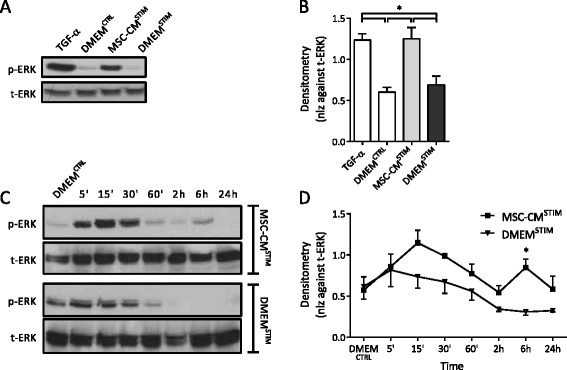
MSC-CM^STIM^ increases ERK1/2 phosphorylation. NCI-H292 cells were incubated with MSC-CM^STIM^, DMEM^STIM^, DMEM^CTRL^ or TGF-α 20 ng/ml (pos ctrl). ERK1/2 phosphorylation and total ERK was determined in cell lysates using Western blot. **a**: After 15 min incubation time MSC-CM^STIM^ increased ERK1/2 phosphorylation compared to its control. **b**: Densitometry for Fig. 3a. Error bars represent SEM, *n* = 6; (*) *p* < 0.05. **c**: Time course experiment, demonstrating that the effect of MSC-CM^STIM^ was most prominent up to 30 min, but could still be observed up to 6 h. DMEM^CTRL^ was obtained at 15 min. **d**: Densitometry for Fig. 3c. Error bars represent SEM, *n* = 4-5; (*) *p* < 0.05

In NCI-H292 monolayers, MSC-CM^STIM^ increased ERK1/2 phosphorylation, when compared to DMEM^STIM^ (Fig. 3a, b). This effect was more pronounced at early time points (15–30 min), but could be observed up to 6 h of stimulation (Fig. 3c, d).

**Fig. 4:**
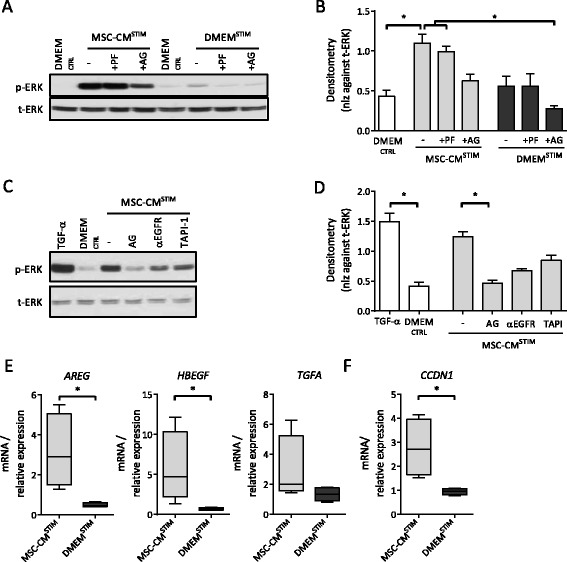
MSC-CM^STIM^ induced ERK1/2 phosphorylation is mediated via (trans)activation of EGFR. **a**: 1 h pre-incubation of NCI-H292 with 0.05 μM PF04217903 and/or 10 μM AG1478, followed by 15 min stimulation with MSC-CM^STIM^ or DMEM^STIM^, or DMEM^CTRL^ showed that inhibition of EGFR decreased ERK1/2 phosphorylation as determined using Western blot. **b**: Densitometry for Fig. 4a. Error bars represent SEM, *n* = 5; (*) *p* < 0.05. **c**: 1 h pre-incubation with 2 μg/ml neutralizing anti-EGFR antibodies and 10 μM TAPI-1 followed by incubation with MSC-CM^STIM^ during 15 min showed that these agents decreased ERK1/2 phosphorylation. **d**: Densitometry for Fig. 4c. Error bars represent SEM, *n* = 3; (*) *p* < 0.05. **e**: NCI-H292 cells were incubated with MSC-CM^STIM^ or DMEM^STIM^ and harvested after 9 h for mRNA analysis. mRNA expression of the EGFR ligands *AREG*, *HBEGF* and *TGFA* was determined by qPCR. MSC-CM^STIM^ significantly increased the expression of *AREG* and *HBEGF*. Expression of *TGFA* increased but this was not significant. **f**: mRNA expression of the proliferation marker *CCDN1* was significantly increased. Values were normalized against *RPL13A* and *ATP5B* reference genes. Box and whiskers represent median, interquartile range and minimum and maximum. *n* = 4; (*) *p* < 0.05

Next, the involvement of two upstream receptors, c-Met (HGF-receptor) and Epidermal Growth Factor Receptor (EGFR), in ERK1/2 phosphorylation by MSC-CM^STIM^ was explored using a tyrosine kinase inhibitor to block c-Met and EGFR, or neutralizing anti-EGFR antibodies. Control experiments showed that ligands for both c-Met (HGF) and EGFR (TGF-α) are potent activators of ERK1/2, and that the respective inhibitors block this activation effectively and specifically (data not shown). At 15 min, phosphorylation of ERK1/2 by MSC-CM^STIM^ was inhibited by the EGFR inhibitor (AG) and EGFR neutralizing antibodies (αEGFR) (Fig. 4a-d), but was not affected by the c-Met inhibitor (PF) (Fig. 4a, b). This shows that although both EGFR and c-Met are potent activators of ERK1/2, the increase in ERK1/2 phosphorylation as observed upon stimulation with MSC-CM^STIM^ is mainly mediated via the EGFR pathway.

Besides direct EGFR-dependent activation of ERK1/2 by constituents of the MSC-CM, it is also known that airway epithelial cells promote wound healing in an autocrine manner, via transactivation of EGFR. In this process, matrix metalloproteinases, predominantly TACE/ADAM17, mediate the shedding of cell surface-bound EGFR ligands [34]. These ligands in turn activate ERK1/2 via EGFR. Addition of a TACE/ADAM17 inhibitor (TAPI-1) decreased ERK1/2 phosphorylation induced by MSC-CM^STIM^ (Fig. 4c, d). This suggests that EGFR transactivation through TACE/ADAM17 contributes to ERK1/2 phosphorylation in NCI-H292 monolayers, in addition to direct effects of MSC-CM^STIM^.

This raised the question whether MSC-CM^STIM^ could induce EGFR ligand expression in airway epithelial cells. To investigate this, NCI-H292 were incubated with MSC-CM^STIM^ and gene expression of several EGFR ligands was assessed. A significant increase of mRNA expression of both *AREG* and *HBEGF* was observed upon stimulation with MSC-CM^STIM^ compared to its control (Fig. 4e).

Enhanced ERK1/2 activation promotes in part cell proliferation by increasing expression of Cyclin D1 (*CCDN1*) [35], a cell cycle regulator. In line with MSC-CM^STIM^ induced ERK1/2 phosphorylation, the mRNA expression of *CCDN1* was significantly higher compared to its control, suggesting an increase in cell proliferation (Fig. 4f).

### Blocking of the EGFR reduces the stimulatory effect of MSC-CM^STIM^ on wound healing

In NCI-H292 monolayers, stimulation with MSC-CM^STIM^ resulted in increased ERK1/2 phosphorylation, and this process appeared to be predominantly regulated via EGFR signaling. To assess if this observation could be translated into a functional effect, wound healing experiments were repeated using the before mentioned tyrosine kinase inhibitors. To limit toxic side effects after prolonged exposure to high doses of AG, we adjusted the concentration of AG to 0.2 μM based on dose-response experiments; for the c-Met inhibitor PF a concentration of 0.05 μM sufficed (data not shown).

**Fig. 5:**
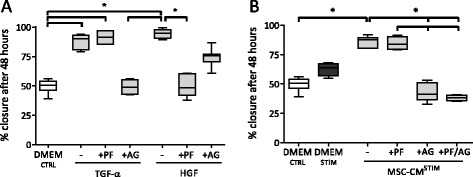
Blocking of EGFR reduces the stimulatory effect of MSC-CM^STIM^ on wound closure. **a**: NCI-H292 wounded cell layers were stimulated with MSC-CM^STIM^ or DMEM^STIM^, DMEM^CTRL^, or DMEM supplemented with TGF-α 20 ng/ml or HGF 20 ng/ml (positive controls). Inhibitors of EGFR (0.2 μM AG1478) or c-Met (0.05 μM PF04217903) were added during the full culture period in assigned conditions. At 48 h, HGF and TGF-α both significantly enhanced wound healing in NCI-H292 cells, which was averted to a level comparable to the negative control by their respective inhibitors. **b**: In the presence of the EGFR inhibitor, the wound healing capacity of MSC-CM^STIM^ decreased to values below those observed in the negative control as determined after 48 h. Inhibition of c-Met alone had no effect on wound healing induced by MSC-CM^STIM^. Box and whiskers represent median, interquartile range and minimum and maximum. *n* = 4-7; (*) *p* < 0.05

The EGFR ligand TGF-α and the c-Met ligand HGF both significantly enhanced wound healing in NCI-H292 monolayers, indicating that both their corresponding receptors could be involved in MSC-mediated wound repair. Inhibition of EGFR as well as of c-Met blocked the effect of TGF-α and HGF, respectively (Fig. 5a). In the presence of the EGFR inhibitor the effect of MSC-CM^STIM^ on wound closure was significantly reduced to a level similar to that observed in the negative control. Blocking of the HGF receptor c-Met alone had no effect on wound closure induced by MSC-CM^STIM^ (Fig. 5b).

These data indicate that signaling through EGFR is the predominant pathway by which MSC-CM^STIM^ increased wound healing in NCI-H292 epithelial cells.

## Discussion

**Fig. 6:**
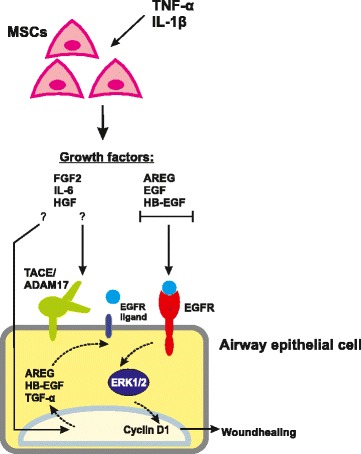
Proposed model of enhanced wound closure in airway epithelial cells by MSC-CM^STIM^. In MSCs, exposure to TNF-α and IL-1β increases the mRNA expression of various growth factors. Conditioned medium from these MSCs contributes to wound healing of airway epithelial cells via three mechanisms: direct activation of EGFR; activation of matrix metalloproteases which results in shedding of membrane bound EGFR ligands; induction of mRNA expression of EGFR ligands by the airway epithelium. Activation of the downstream MAP kinase ERK1/2, which is known as a regulator of cell proliferation (a.o. assessed by increased Cyclin D1) and differentiation, subsequently results in increased wound healing

This study shows for the first time that stimulation of MSCs with pro-inflammatory cytokines improves their capacity to enhance airway epithelial repair in an *in vitro* repair model using the airway epithelial cell line NCI-H292. We show that conditioned medium from human bone marrow-derived MSCs increases wound healing in airway epithelial cells and that this effect is significantly enhanced when the MSCs are treated with a mixture of pro-inflammatory cytokines, i.e. TNF-α and IL-1β. These cytokines increased the mRNA expression of the growth factors *FGF2, HBEGF*, *HGF*, and of *IL6* in MSCs. We provide evidence for the possible involvement of the following mechanisms in this enhancing effect of MSC-CM^STIM^ on wound repair (Fig. 6): first, MSC-CM^STIM^ directly activated the MAP kinase ERK1/2 via EGFR, resulting in wound healing. Second, MSC-CM^STIM^ caused ADAM-mediated transactivation of EGFR, further contributing to wound healing. Third, MSC-CM^STIM^ increased mRNA expression of EGFR ligands *AREG* and *HBEGF* in NCI-H292 airway epithelial cells.

Previous studies show that MSCs contribute to epithelial repair in wound healing models in vivo as well as *in vitro* [8, 17, 36], and that pro-inflammatory cytokines can attract MSCs to sites of inflammation [37]. Within such an inflammatory environment, MSCs display an anti-inflammatory phenotype that is characterized by an increased expression of *IL6* [38]. In line with this observation, we noted increased expression of *IL6* in MSCs stimulated with TNF-α and IL-1β, suggesting anticipated response to these pro-inflammatory stimuli. Besides increased mRNA expression of *IL6,* we observed increased mRNA expression of several growth factors in TNF-α/IL-1β stimulated MSCs, suggesting that the regenerative potential of these MSCs is enhanced. The increased mRNA expression of several EGFR ligands was not accompanied by increased levels of these ligands as detected by ELISA (data not shown). This may be explained by limitations in the sensitivity of the ELISAs, rapid binding of secreted growth factors to their cellular receptors, and/or by the fact that the observed effects are not explained by detectable levels of single EGFR ligands, but by synergisms between various released mediators. In line with our observations, previous reports also demonstrated that stimulation with pro-inflammatory cytokines induces growth factor expression by MSCs [20, 39, 40]. However, to our knowledge this is the first study demonstrating that pro-inflammatory cytokine stimulation of MSCs induces wound healing in airway epithelial cells, and our data indicate that this may involve the action of growth factors. Together, this suggests that the capacity of MSCs to enhance wound repair may be increased upon recruitment to areas of inflammation where they are exposed to pro-inflammatory cytokines [25]. This is relevant for a disease such as COPD, where both inflammation and tissue damage are present.

Amongst the growth factors induced in TNF-α/IL-1β-stimulated MSCs, EGFR ligands and HGF are involved in airway epithelial wound repair [41–44]. By using inhibitors for EGFR and c-Met, we show that the effect of MSC-CM^STIM^ on both ERK1/2 activation as well as on wound healing was mediated by EGFR activation, without an apparent effect of c-Met inhibition. As it has been shown that HGF can promote airway epithelial wound repair [43, 44], we speculate that in our model HGF has a more subtle contribution to wound healing that is masked by the major role of EGFR signaling in airway epithelial repair. This is supported by a study from Curley et al., who showed that the addition of HGF-neutralizing antibodies to MSC-CM in a scratch wound assay using A549 alveolar epithelial cells did not affect wound repair [45]. Besides HGF and EGFR ligands, other MSC-CM^STIM^ constituents can contribute to wound healing. One example of a possibly involved MSC-derived mediator is IL-6, which was previously found to contribute to MSC-mediated epithelial wound repair [36, 46]. Although in our model blocking of EGFR fully inhibited the stimulatory effect of MSC-CM^STIM^, we cannot exclude a contribution of MSC-derived IL-6.

In addition to direct EGFR activation, we observe that MSC-CM^STIM^ activates ERK1/2 via transactivation of EGFR. EGFR-transactivation results from activation of G-protein coupled receptors (GPCRs) that activate proteases of the ADAM family, such as TACE/ADAM17. These proteases cleave cell surface-bound EGFR ligands, resulting in autocrine EGFR activation [34]. Numerous factors are able to activate GPCRs, and our study focussed on the role of a modest selection of growth factors and chemokines. It was beyond the scope of this study to investigate other ligands potentially released by MSCs, but this will be an interesting point for future investigations.

In our model, we have used the airway epithelial cell line NCI-H292. This cell line has been shown to respond in a similar fashion as primary epithelial cells of the lung and is frequently used to study effects mediated by the EGFR axis [47–50]. The use of a cell line limits translation to the in vivo situation, where in addition to the epithelium also immune and endothelial cells interact in the process of wound repair. The benefit on the other hand is that it allows for detailed investigation of MSC-CM^STIM^ effects on the ERK1/2 signaling pathway as well as on cell proliferation.

The circular wounds used in this study are relatively large and NCI-H292 cells require cell proliferation in order to close this type of wound as we have shown previously [32]. This adds information about effects on cell proliferation that cannot be obtained when using primary bronchial epithelial cells (PBEC) using wounds of a similar size, as migratory mechanisms appear to suffice for the closure of this type of wounds [51]. For the same reasons, the wound repair model also provides additional information to the more commonly used scratch wound assays, as these scratch wounds close quickly, and primarily via cell migratory mechanisms [17]. Using ALI-PBEC, we observed effects of MSC-CM^STIM^ on epithelial wound healing, but unlike the observation in NCI-H292, this effect was not significantly different compared to control medium (DMEM^STIM^). We speculate that higher intrinsic rate of wound closure in ALI-PBEC and donor variability limited the experimental window to observe beneficial effects of MSC-CM. Besides, differences in intrinsic wound healing characteristics (e.g. primarily via migration rather than proliferation in ALI-PBEC), as well as direct effects of TNF-α and IL-1β on migratory processes might further explain differences in effects of MSC-CM^STIM^ on ALI-PBEC versus NCI-H292.

Our data provide evidence for the concept of a cell-based therapy with cells that specifically interact with an inflammatory environment to enhance their beneficial properties. Safety concerns regarding tumorigenesis or fibrosis might arise when using cell-based therapies, but based on current data obtained from clinical trials the use of MSCs in patients is considered to be safe [52]. Moreover, it has been shown in multiple studies that MSCs can induce apoptosis of cancer (but not healthy) cells via tumor necrosis factor-related apoptosis-inducing ligand [53, 54].

MSCs are currently considered as treatment for COPD and the first patient safety and feasibility study has recently been published [55]. Interestingly, the results from the present study suggest that exposure of MSCs to pro-inflammatory cytokines increases their ability to repair damaged tissue. This may imply that MSCs are more effective at sites of inflammation and that* in vitro* stimulation of MSCs with cytokines before infusion in patients may potentially result in a larger therapeutic effect. Future investigations should be directed at further exploring the mechanisms involved in the enhancing effect of MSCs on epithelial wound healing, using e.g. primary airway and alveolar epithelial cells, preferably using co-cultures of MSCs with epithelial cells.

## Conclusions

We have found that MSC conditioned medium obtained from MSCs stimulated with a mixture of cytokines potently enhances wound repair in injured airway epithelial cells. This effect is predominantly mediated by (trans)activation of EGFR and subsequent activation of the ERK1/2 signaling cascade. This observation implies that in areas of tissue damage where inflammatory mediators are present (such as in the lungs of COPD patients), MSCs may display increased regenerative properties via the secretion of growth factors. This supports the concept that MSCs are a promising candidate for cell-based therapy in inflammatory lung diseases such as COPD.

## Abbreviations

ADAM: a disintegrin and metalloprotease
ALI: air liquid interface
AREG: amphiregulin
BSA: bovine serum albumin
BPE: bovine pituitary extract
CCDN1: cyclin D1
CM: conditioned medium
COPD: chronic obstructive pulmonary disease
EGF(R): epidermal growth factor (receptor)
ERK: extracellular signal-regulated kinase
FGF2: fibroblast growth factor 2
GPCR: G-protein coupled receptor
HB-EGF: heparin binding EGF-like growth factor
HC: hydrocortisone
HGF: hepatocyte growth factor
IL-1β: interleukin-1β
IL-6: interleukin-6
MAPK: mitogen activated protein kinase
MMP: matrix metalloproteinase
MSC: mesenchymal stromal cell
MSC-CM^CTRL^: conditioned medium obtained from unstimulated MSCs
MSC-CM^STIM^: conditioned medium obtained from TNF-α/IL-1β stimulated MSCs
PBEC: primary bronchial epithelial cells
PDGFA: platelet derived growth factor a
RA: retinoic acid
SEM: standard error of the mean
TACE: TNF-α converting enzyme
TGF-α: transforming growth factor-α
TNF-α: tumour necrosis factor-α
VEGF(R): vascular endothelial growth factor (receptor)
